# Flavonoid-Rich Ethanol Extract from the Leaves of *Diospyros kaki* Attenuates D-Galactose-Induced Oxidative Stress and Neuroinflammation-Mediated Brain Aging in Mice

**DOI:** 10.1155/2018/8938207

**Published:** 2018-12-23

**Authors:** Yingjuan Ma, Bin Ma, Yuying Shang, Qingqing Yin, Dejie Wang, Song Xu, Yan Hong, Xunyao Hou, Xueping Liu

**Affiliations:** ^1^Department of Senile Neurology, Shandong Provincial Hospital Affiliated to Shandong University, Jinan, 250021 Shandong, China; ^2^School of Pharmaceutical Sciences of Shandong University, Jinan, 250012 Shandong, China; ^3^Department of Critical Care Medicine, Jining No. 1 People's Hospital, Jining 272011, China; ^4^Corporate Health Management Center, Shandong Electric Powder Central Hospital, Jinan, 250001 Shandong, China; ^5^Department of Anti-Aging, Shandong Provincial Hospital Affiliated to Shandong University, Jinan, 250021 Shandong, China; ^6^Anti-Aging Monitoring Laboratory, Shandong Provincial Hospital Affiliated to Shandong University, Jinan, 250021 Shandong, China

## Abstract

Aging is a major factor that contributes to neurological impairment and neuropathological changes, such as inflammation, oxidative stress, neuronal apoptosis, and synaptic dysfunction. Flavonoids act as protective antioxidant and anti-inflammatory agents against various age-related neurodegenerative diseases. Here, we investigated the protective effect and mechanisms of the flavonoid-rich ethanol extract from the leaves of *Diospyros kaki* (FELDK) in the cortex and hippocampus of D-galactose- (gal-) aged mice. Our results showed that FELDK treatment restored memory impairment in mice as determined by the Y-maze and Morris water maze tests. FELDK decreased oxidative stress levels via inhibiting reactive oxygen species (ROS) and malondialdehyde (MDA) production and elevating antioxidative enzymes. FELDK also alleviated D-gal-induced neuroinflammation via suppressing the expression of advanced glycation end products (AGEs) and receptor for AGEs (RAGE) and activating microgliosis and astrocytosis, nuclear factor kappa B (NF-*κ*B) nuclear translocation, and downstream inflammatory mediators. Moreover, FELDK inhibited the phosphatidylinositol 3-kinase (PI3K)/Akt and C-jun N-terminal kinase (JNK) apoptotic signaling pathways and ameliorated the impairment of synapse-related proteins. Hence, these results indicate that FELDK exerts neuroprotective effects on D-gal-induced brain aging. Thus, FELDK may be a potential therapeutic strategy for preventing and treating age-related neurodegenerative diseases such as Alzheimer's disease.

## 1. Introduction

Aging is a major factor underlying brain dysfunction, which is marked by memory loss, cognitive impairment, and many age-related neurodegenerative disorders, such as Alzheimer's disease (AD) [1]. Multidimensional pathological features, including oxidative stress, neuroinflammation, and cell metabolic imbalance, are associated with aging of the brain [2, 3], which consequently result in neuronal death and synaptic dysfunction. Oxidative stress and reactive oxygen species (ROS) are known contributors to age-related neurodegenerative disorders [4, 5]. The accumulation of excessive oxidative stress and ROS in the brain can trigger neuroinflammation and induce dangerous modifications of cellular proteins, lipids, and DNA, which subsequently impair cellular activity and destabilize neuronal homeostasis, ultimately leading to neuronal loss [1, 5, 6].

Chronic inflammation is a potential risk factor in age-associated diseases [7]. D-Galactose (D-gal) can mimic aging in animal models by inducing oxidative stress and inflammation, and these models have been widely used in antiaging research [8–11]. Accumulation of D-gal triggers ROS generation, which leads to oxidative stress and formation of advanced glycation end products (AGEs) via reacting with amino peptides and proteins [12]. In addition, binding of AGEs to the receptor for advanced glycation end products (RAGE) is common in many age-related degenerative disorders. This interaction leads to downstream activation of nuclear factor kappa B (NF-*κ*B), followed by release of ROS and proinflammatory mediators [13]. Evidence indicates that ROS and AGEs result in neuroinflammation, activation of microglia and astrocytes, loss of neuronal cells, and reduction of synapse-related proteins, which can aggravate the deficits of learning and memory [6, 14, 15]. Thus, we used D-gal-induced aging mice in this study to study the aging brain.

Extracts from persimmon leaves, of which flavonoid is the main component, have been widely used in China to treat apoplexy and its sequela [16]. In addition to its well-known antioxidant and neuroprotective effects, this leaf extract also provides a number of other beneficial aspects. Studies reported that a standardized flavonoid extract of persimmon leaves (FLDK) exerted protective effects *in vivo* in two different brain ischemia and reperfusion injury rat models, as well as protective effects *in vitro* in hippocampal and primary cortical neurons following glutamate or hypoxia injury [16]. FLDK also protected NG108-15 cells from oxidative injury induced by hydrogen peroxide, possibly by improving the cellular redox state and upregulating Bcl-2 levels [17]. Ethyl acetate extract from persimmon leaves showed a potential protective effect on A*β*_1-42_-induced cognitive dysfunction in rats, probably via improving the antioxidative defense system and attenuating mitochondrial-mediated neuronal apoptosis. Flavonoids and triterpenoids are likely the major active components in the ethyl acetate extract [18]. However, it is unclear if the flavonoid-rich ethanol extract from the leaves of *Diospyros kaki* (FELDK) could delay aging. Therefore, the present study is aimed at investigating the neuroprotective effects of FELDK on D-gal-induced learning and memory impairment, neuroinflammation, oxidative stress, apoptosis, and synaptic function.

## 2. Materials and Methods

### 2.1. Reagents

FELDK was provided by Professor Bin Ma (School of Pharmaceutical Sciences of Shandong University). FELDK was preparated as we previously described [19]. High-performance lipid chromatography (HPLC) analyses were carried out using an Agilent 1200 series including a binary pump, a column oven, a 4-flow channel degasser (Agilent Technologies, Palo Alto, CA, USA), and an autosampler (NASCA 5100, Shiseido Co. Ltd., Tokyo, Japan). FELDK were separated on a Phenomenex Luna C_18_ column (5 *μ*m particle size, 250 mm × 4.6 mm i.d. with a 4 mm × 3 mm precolumn (Security Guard C18 cartridge, Phenomenex Inc., USA)). The mobile phases were composed of acetonitrile (A) and water with 0.1% formic acid (B) using a multistep gradient elution of 15% A for 0–10 min, 35% A for 10–20 min, 95% A for 20–45 min, 95% A for 45–55 min, 15% A for 55–55.1 min, and 15% A at 55.10–60.0 min with the flow rate kept at 1.0 mL/min. The injected sample volume was set at 5 *μ*L, and the column oven temperature was 20°C.

After separation, the HPLC flow was directed to the mass spectrometer (MS) inlet after 1 : 10 splitting. FELDK analyses were performed on an API 4000 mass spectrometer (Applied Biosystems Sciex, Ontario, Canada) with an electrospray ionization source (ESI) in negative mode. Nitrogen was used as the carrier, heater, and collision gases. The Applied Biosystems Analyst version 1.5.2 software was used to control for data acquisition and analysis [20].

HPLC-MS verified that the ethanol extract from the leaves of *Diospyros kaki* contained the following flavonoids: myricetin, quercetin, kaempferol, hyperoside, astragalin, and vitexin [18, 21]. After being hydrolyzed to aglycone, the extract was contained more than 79.35% total flavonoids as determined by UV spectrophotometry as previously described [22].

Enzyme-linked immunosorbent assay (ELISA) kits for mouse AGEs were from Cell Biolabs (San Diego, CA, USA). The RAGE antibody was from Santa Cruz Biotechnology (CA, USA), NF-*κ*B p65 antibody was from Millipore (Temecula, CA, USA), and tumor necrosis factor-*α* (TNF-*α*) and interleukin-1*β* (IL-1*β*) antibodies were purchased from ImmunoWay Biotechnology (Newark, DE, USA). The phosphatidylinositol 3-kinase (PI3K), phosphorylated- (p-) Akt, total- (t-) Akt, p-C-jun N-terminal kinase (JNK), and t-JNK antibodies were purchased from Cell Signaling Technology (Beverly, MA, USA). Glial fibrillary acidic protein (GFAP), ionized calcium-binding adaptor molecule (Iba1), Bcl-2, Bax, synaptophysin, synaptotagmin, p-cAMP response element-binding protein (CREB), t-CREB, and p-Ca^2+^/calmodulin-dependent protein kinase II (CaMKII) antibodies were from Abcam (Burlingame, CA, USA). The Iba1 antibody used for immunohistochemical staining was from Wako Pure Chemical Industries (Osaka, Japan). The histone H3, cyclooxygenase-2 (COX-2), and inducible nitric oxide synthase (iNOS) antibodies were from Proteintech Group (Chicago, IL, USA). Glyceraldehyde phosphate dehydronase (GAPDH) and secondary antibodies were from Zhongshan Jinqiao Biotechnology (Beijing, China). The BCA Protein Assay Kit was from Biocolor BioScience & Technology Company (Shanghai, China).

### 2.2. Animals and Treatment

Thirty-six 10-week-old Kunming male mice (purchased from the Experimental Animal Center of Shandong University, Jinan, China) were housed in a constant environment (12 h/12 h light-dark schedule; 19–23°C; 45–65% humidity) with free access to water and food. After feeding adaptation for one week, the mice were randomly divided into four groups (*n* = 9 per group). The aging model group (D-gal group) received subcutaneous injections of D-galactose at 100 mg/(kg d) for 10 weeks to mimic aging and was orally gavaged with vehicle. The normal control group (NC group) were injected with normal saline and received the same volume of vehicle by oral gavage. The flavonoids F1 and F2 intervention groups were fed 40 and 80 mg/(kg d) FELDK, respectively, by oral gavage after subcutaneous injection of D-gal. The NC group and D-gal group were given the same volume of normal saline. Animal breeding and all experiments complied with the Provisions and General Recommendation of the Chinese Experimental Animals Administration Legislation, and the experiments were approved by the Animal Care Committee of the Provincial Hospital Council, Shandong University, China.

### 2.3. Behavioral Tests

After 10 weeks of oral FELDK treatment, the Y-maze test and the Morris water maze (MWM) test were performed for behavioral analysis. The two tests were separated by one day.

#### 2.3.1. Y-Maze Test

The Y-maze test was performed as previously described to determine working memory performance [6, 9]. After being acclimated in the environment, each mouse was placed at the end of one arm of the maze. The series of arm entries were recorded during three 8 min sessions. Spontaneous alteration behavior was defined as the number of entries into three arms. Alteration behavior (%) was calculated as follows: (alterations/total entries − 2) × 100.

#### 2.3.2. MWM

Two days after the Y-maze test, the MWM test was used to assess learning and memory in the mice. The MWM task consisted of a five-day training period followed by a one-day probe test. The MWM device was a circular tank (120 cm in diameter and 50 cm in height) with a platform (10 cm in diameter) situated in the center of one of the four equally sized quadrants and submerged ~1 cm under water (22 ± 1°C) during the training period. The platform was removed on the last day of the probe test. On training days, mice were placed into one of the four quadrants. Escape latency (EL) was measured as the time that mice spent discovering the platform. Mice that failed to find the platform were assisted to the platform and kept on the platform for 15 sec. For these mice, EL was recorded as 60 sec. In the probe trial, mice were allowed to swim freely for 60 sec. EL, the number of times mice crossed the platform area, and the time spent in the target quadrant were counted. All data were recorded by a computerized video recording system (HVS Image, UK) with a video camera located above the center of the pool.

### 2.4. Preparation of Tissue Samples

During the experiment, three mice (one in the D-gal group, one in the F1 group, and one the in F2 group) died and were excluded from data analysis. After the MWM test, all mice were anesthetized and perfused transcardially with 30 mL normal saline. The left hemisphere of the mice brains was soaked in 4% paraformaldehyde for 48 h, followed by postfixation in 70% ethanol for 24 h, and then dehydrated and embedded in molten paraffin. The brains were sliced coronally into 5 *μ*m thick serial sections for immunohistochemistry. The right cortex and hippocampus of hemispheres were dissected and stored in liquid nitrogen; parts were used for biochemical detection, and the others were used for western blot analysis.

### 2.5. Tissue Homogenates

For biochemical assays, tissues were homogenized in 1/10 (*w*/*v*) normal saline containing a protease inhibitor and phosphatase inhibitor (Sigma-Aldrich) and then centrifuged at 12000 g at 4°C for 10 min. The supernatants were collected and stored at −80°C. For western blot analysis, brains were homogenized in RIPA lysis buffer (1 : 4, *w* : *v*) containing 1% protease inhibitors and 1% phosphatase inhibitors (Sigma-Aldrich) and then centrifuged at 12000 g at 4°C for 30 min. Nuclear protein was extracted to measure NF-*κ*B p65 expression according to the manufacturer's instructions (Thermo Scientific, Waltham, MA). Protein concentration was quantified using the BCA Protein Assay Kit.

### 2.6. Biochemical Analysis

#### 2.6.1. ELISA for AGEs

Brain AGEs levels were detected using the OxiSelect™ AGEs ELISA kit according to the manufacturer's instructions. Absorbance at 450 nm was measured, and content was expressed as ng/mg protein.

#### 2.6.2. Measurement of Oxidative Stress

ROS and malondialdehyde (MDA) levels, as well as superoxide dismutase (SOD), glutathione peroxidase (GSH-Px), and catalase (CAT) activity, were detected as previously published [23]. ROS was measured by determining the oxidation levels of 2′,7′-dichlorodihydrofluorescein-diacetate compared to 2′,7′-dichlorofluorescein-diacetate. MDA levels were estimated using the thiobarbituric acid reactive method. SOD activity was measured by the inhibition of nitro blue tetrazolium reduction. GSH-Px activity was determined by 5,5′-dithiobis-p-nitrobenzoic acid. CAT activity was assayed as the rate of reduction in absorbance of H_2_O_2_ at 240 nM/min/mg protein in the presence of CAT.

### 2.7. Western Blotting

Equal amounts of protein were separated on SDS-PAGE gels and then transferred to PVDF membranes. Membranes were incubated with antibodies against RAGE (1 : 500), GFAP (1 : 1000), Iba1 (1 : 1000), NF-*κ*B (1 : 1000), TNF-*α* (1 : 1000), IL-1*β* (1 : 1000), COX-2 (1 : 1000), iNOS (1 : 1000), PI3K (1 : 1000), p-Akt (1 : 2000), t-Akt (1 : 1000), p-JNK (1 : 1000), t-JNK (1 : 1000), Bcl-2 (1 : 500), Bax (1 : 1000), synaptophysin (1 : 20000), synaptotagmin (1 : 1000), p-CREB (1 : 5000), t-CREB (1 : 1000), p-CaMKII (1 : 1000), GAPDH (1 : 1000), and histone H3 (1 : 1000), followed by the appropriate corresponding secondary antibody (1 : 5000) for 1 h at room temperature. Proteins were visualized with enhanced chemiluminescence reagents using an image analyzer (Alpha Innotech, San Leandro, CA, USA). Protein bands were quantified using ImageJ (NIH, USA).

### 2.8. Immunohistochemistry

Brain sections were dewaxed, rehydrated, and boiled in citrate buffer (10 mM, pH 6.0) for antigen retrieval, followed by incubation with 0.3% H_2_O_2_ and then goat serum. Primary rabbit anti-Iba1 (1 : 200) and rabbit anti-GFAP (1 : 200) antibodies were incubated overnight at 4°C. After repeated washing, the slices were then incubated with biotinylated goat anti-rabbit IgG followed by incubation with horseradish perioxidase (HRP) conjugated streptavidin. Finally, the slices were incubated with DAB and counterstained with hematoxylin.

### 2.9. Statistical Analysis

Statistical analysis was performed using GraphPad Prism version 6.01 (GraphPad Software Inc., La Jolla, CA, USA). Significant differences between treatment groups were analyzed with one-way analysis of variance (ANOVA) followed by Tukey's test. Repeated measures ANOVA followed by Tukey's test was used for comparison of escape latency in the MWM test. Data are expressed as means ± standard error of the mean (SEM). *P* values < 0.05 were considered statistically significant.

## 3. Results

### 3.1. Effects of FELDK on Memory Impairment in D-gal-Aged Mice

**Figure 1:**
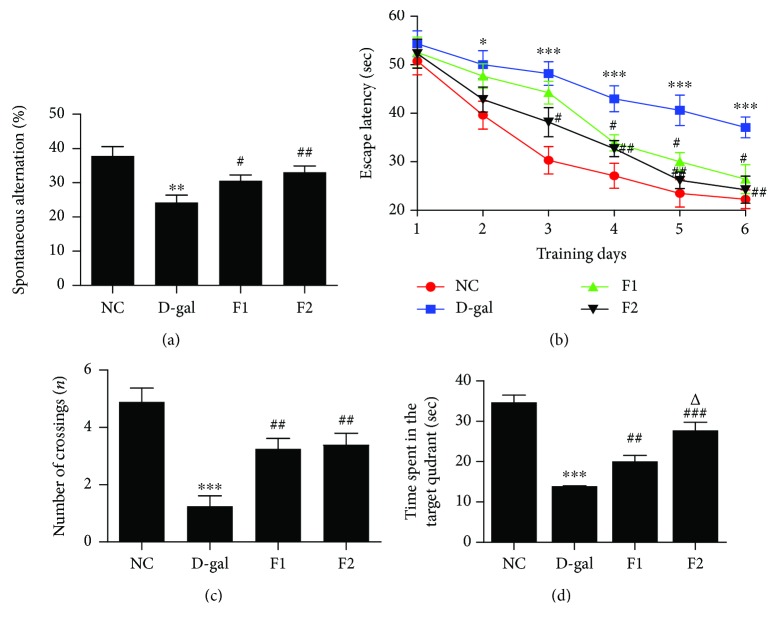
Effects of FELDK on memory impairment in D-gal-treated mice. (a) The percentage of spontaneous alteration in the Y-maze test. (b) Comparison of EL during 6 days in the MWM. (c) The number of crossings and (d) the time spent in the target quadrant where the platform had been previously situated during the spatial probe trial on day 6. Data are presented as mean value ± SEM for *n* = 9, 8, 8, 8. ^∗^*P* < 0.05, ^∗∗^*P* < 0.01, ^∗∗∗^*P* < 0.001 versus NC group; ^#^*P* < 0.05, ^##^*P* < 0.01, ^###^*P* < 0.001 versus the D-gal group.

The Y-maze was used to assay spatial working memory in mice. The D-gal group showed a significant decrease in the percentage of spontaneous alteration compared to the NC group (*P* < 0.01) (Figure 1(a)), indicating decreased working memory in the D-gal-aged mice. The percentage of spontaneous alteration was significantly increased in FELDK-treated mice at both 40 and 80 mg/kg (*P* < 0.05 and *P* < 0.01, respectively), indicating that FELDK improves working memory in D-gal-aged mice.

The MWM test was used to measure the effects of FELDK on spatial learning and memory. As shown in Figure 1(b), EL increased in the D-gal group on the second day compared to the NC group (*P* < 0.05). Administration of oral FELDK (40 and 80 mg/kg) significantly reduced EL (both *P* < 0.05). In the probe test (Figures 1(c) and 1(d)), the searching frequency (the number of times that the mice crossed the site where the platform had been placed) and the time spent in target quadrant (the swimming time in the site where the platform had been placed) decreased in the D-gal group compared to the NC group (both *P* < 0.001). FELDK at both dosages (40 and 80 mg/kg) alleviated the decreased number of crossings and the time spent in target quadrant (*P* < 0.01). In addition, the high dose of FELDK (80 mg/kg) showed better effects than the low dose of FELDK (40 mg/kg) on the time spent in the quadrant (*P* < 0.05). These results suggest that FELDK alleviates spatial learning and memory impairment in D-gal-aged mice.

### 3.2. Effects of FELDK on AGEs and RAGE Levels in D-gal-Aged Mice

**Figure 2:**
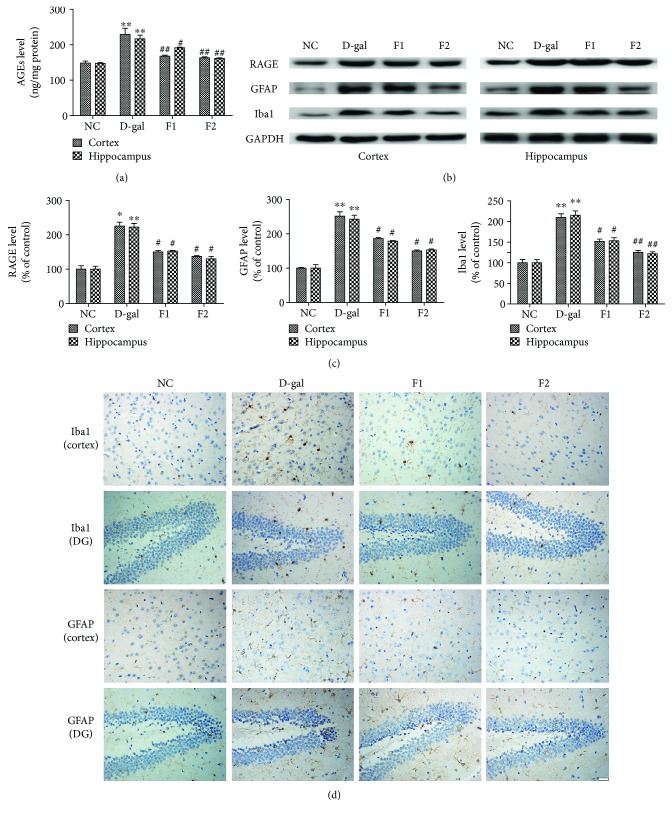
Effects of FELDK on AGEs, RAGE, GFAP, and Iba1 expression in the cortex and hippocampus of D-gal-aged mice. (a) AGEs levels were determined by ELISA (*n* = 9, 8, 8, 8). (b) Representative western blots showing protein expression of RAGE, GFAP, and Iba1 (*n* = 3). (c) Quantification of western blot analysis of RAGE, GFAP, and Iba1 protein expressed as percent of control. (d) The immunohistochemical images of active microglia and astrocytes in the cortex and hippocampal dentate gyrus (DG) regions of mice (*n* = 9, 8, 8, 8). Scale bars = 50 *μ*m. All data are presented as mean values ± SEM. ^∗^*P* < 0.05, ^∗∗^*P* < 0.01 versus the NC group; ^#^*P* < 0.05, ^##^*P* < 0.01 versus the D-gal group.

As shown in Figure 2(a), D-gal significantly (*P* < 0.01) increased AGEs levels in the hippocampus and cortex regions, which was attenuated by FELDK (*P* < 0.05). RAGE is a cell surface protein that is a member of the immunoglobulin superfamily. Our results showed that the interaction of D-gal with its receptor, RAGE, significantly increased RAGE expression in the hippocampus and cortex regions compared to the NC group (*P* < 0.01). However, FELDK markedly reversed this upregulation of RAGE compared to the D-gal group (*P* < 0.05) (Figures 2(b) and 2(c)).

### 3.3. FELDK Suppressed Microglial and Astrocytes in D-gal-Aged Mice

Glial cells have been considered the major pathological contributor to neurodegeneration [6]. Iba1 and GFAP are specific markers of microglia and astrocyte activation, respectively. A previous study demonstrated that D-gal activates glial cells [6]. As shown in Figures 2(b) and 2(c), western blots showed significant increases in GFAP and Iba1 protein expression in the D-gal group compared to the NC group (*P* < 0.01), and these expression levels were attenuated by low and high doses of FELDK (*P* < 0.05). Similar to the western blotting results, immunohistochemistry showed an increase in activated Iba1^+^ and GFAP^+^ cells in the cortex and hippocampal dentate gyrus (DG) regions of the D-gal vehicle group compared to the vehicle NC group (Figure 2(d)). FELDK treatment decreased the number of activated microglia and astrocytes in the cortex and hippocampal dentate gyrus regions of D-gal-FELDK-cotreated mice.

### 3.4. FELDK Reduced Nuclear NF-*κ*B Translocation and Various Inflammatory Markers in D-gal-Aged Mice

**Figure 3:**
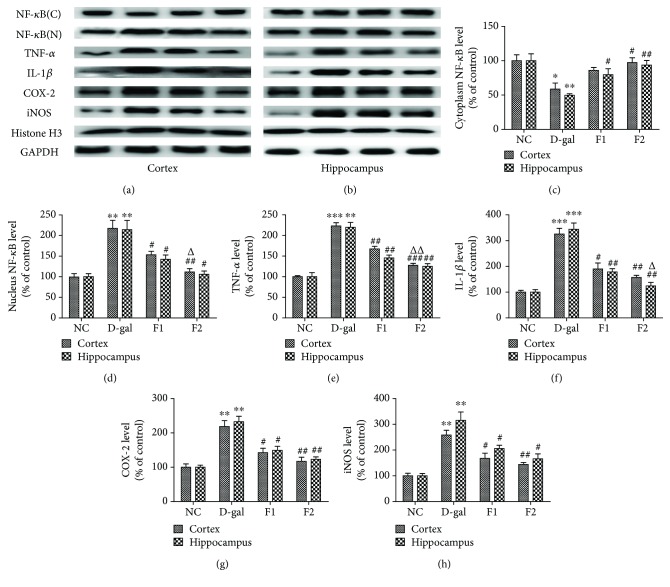
Effects of FELDK on NF-*κ*B nuclear translocation and expression of inflammatory markers in the cortex and hippocampus of D-gal-aged mice. (a) Western blot images showing the expression of cytoplasmic and nuclear NF-*κ*B, TNF-*α*, IL-1*β*, COX-2, and iNOS protein levels. (b) Quantification of the western blot analysis of cytoplasmic and nuclear NF-*κ*B, TNF-*α*, IL-1*β*, COX-2, and iNOS protein expressed as percent of control. All data are presented as mean values ± SEM for *n* = 3. ^∗^*P* < 0.05, ^∗∗^*P* < 0.01, ^∗∗∗^*P* < 0.001 versus the NC group; ^#^*P* < 0.05, ^##^*P* < 0.01, ^###^*P* < 0.001 versus the D-gal group; ^△^*P* < 0.05, ^△△^*P* < 0.01 versus the F1 group.

AGEs promote proinflammatory cytokines through RAGE/NF-*κ*B signaling [15]. To confirm this finding, we measured nuclear and cytoplasmic NF-*κ*B levels. As shown in Figure 3, subcutaneous injection of D-gal significantly increased nuclear translocation of NF-*κ*B (cytoplasm: *P* < 0.05; nucleus: *P* < 0.01) compared to the NC group. FELDK administration attenuated nuclear translocation (nucleus *P* < 0.05; cytoplasm *P* < 0.05). Nuclear translocation of NF-*κ*B increased the expression of proinflammatory cytokines. In this study, the D-gal group mice showed a significant increase in inflammatory biomarkers, including TNF-*α*, IL-1*β*, COX-2, and iNOS (*P* < 0.01, *P* < 0.001, *P* < 0.01, and *P* < 0.01, respectively). Cotreatment with FELDK at both low and high doses attenuated neuroinflammatory cytokine production (TNF-*α*, IL-1*β*, COX-2, and iNOS) compared to the D-gal group (*P* < 0.05).

### 3.5. FELDK Attenuates Oxidative Stress in D-gal-Aged Mice

**Figure 4:**
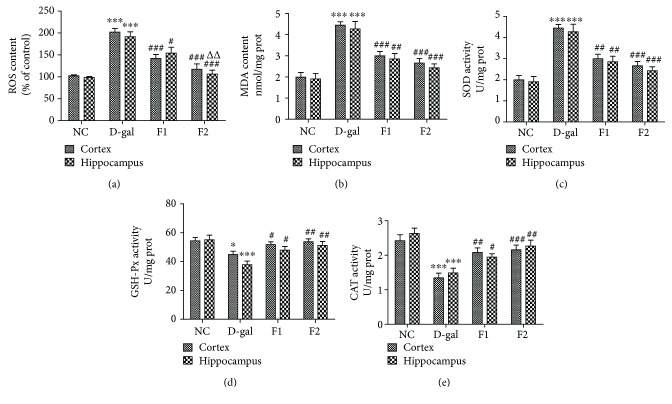
Effects of FELDK on ROS and MDA production and SOD, GSH-Px, and CAT activity in the cortex and hippocampus of D-gal-aged mice. (a) ROS levels. (b) MDA levels. (c) SOD activity. (d) GSH-Px activity. (e) CAT activity. All data are presented as mean values ± SEM for *n* = 9, 8, 8, 8. ^∗^*P* < 0.05, ^∗∗∗^*P* < 0.001 versus the NC group; ^#^*P* < 0.05, ^##^*P* < 0.01, ^###^*P* < 0.001 versus the D-gal group, ^△△^*P* < 0.01 versus the F1 group.

Oxidative stress is initiated early in neurodegenerative diseases, such as AD. Therefore, we measured ROS production to determine the level of oxidative stress in the aged mice brains. D-gal markedly increased ROS production compared to the NC group (*P* < 0.001), while cotreatment of D-gal and FELDK significantly alleviated ROS production (*P* < 0.05) (Figure 4(a)). MDA is a marker of lipid peroxidation. As shown in Figure 4(b), there was a marked increase in MDA in the D-gal-aged mice (*P* < 0.001), while FELDK treatment significantly reduced MDA levels in the F1 and F2 groups (*P* < 0.01). Additionally, SOD, GSH-Px, and CAT activity were measured to determine the antioxidant capacity of the aged brains (Figures 4(c)–4(e)). All of the above markers were reduced after D-gal administration (*P* < 0.001, *P* < 0.05, and *P* < 0.001, respectively), while FELDK significantly improved SOD, GSH-Px, and CAT activity (*P* < 0.05).

### 3.6. FELDK Alleviates Neuronal Apoptosis in the Brains of D-gal-Aged Mice

**Figure 5:**
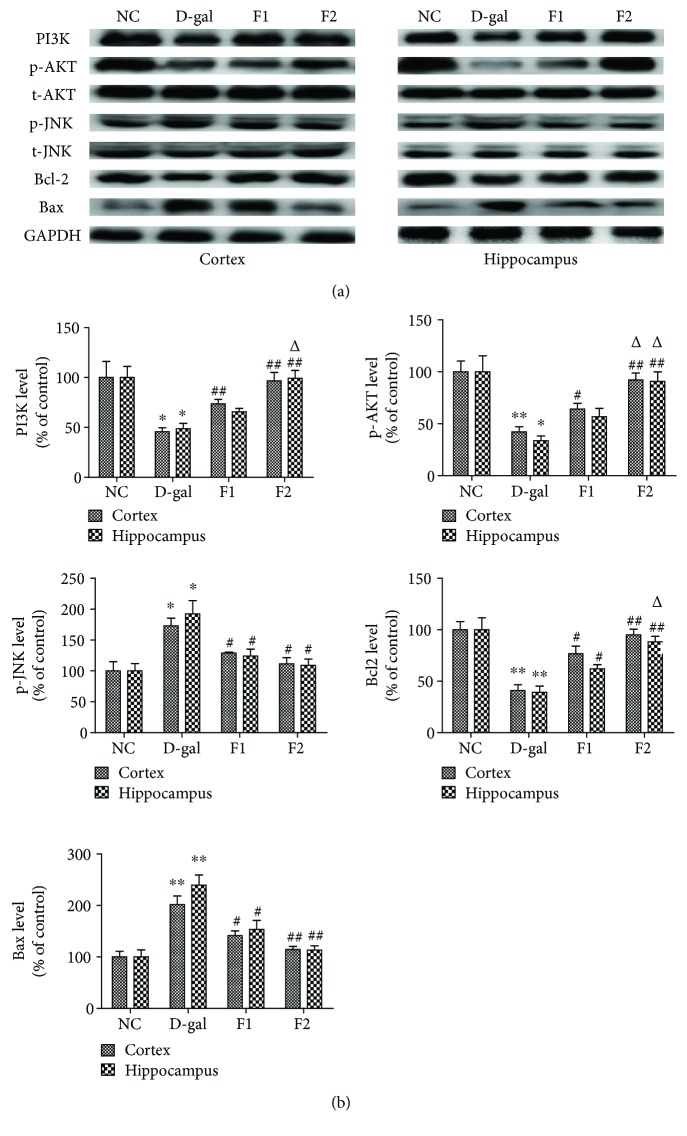
Effects of FELDK on the PI3K-Akt pathway, JNK activation, and Bcl-2 and Bax expression in the cortex and hippocampus of D-gal-aged mice. Western blots and related quantification of PI3K, p-Akt (Ser473), p-JNK, Bcl-2, and Bax expressed as percent of control. All data are presented as mean values ± SEM for *n* = 3. ^∗^*P* < 0.05, ^∗∗^*P* < 0.01 versus the NC group; ^#^*P* < 0.05, ^##^*P* < 0.01 versus the D-gal group; ^△^*P* < 0.05 versus the F1 group.

D-gal has been reported to cause neuronal apoptosis [14]. The PI3K/Akt signaling axis is a major signaling pathway that promotes cell survival. Thus, we used western blot analysis to measure the effects of FELDK on D-gal-induced neuronal apoptosis. The results showed that PI3K and p-Akt decreased markedly in the cortex and hippocampus of D-gal-aged mice compared to the NC mice (*P* < 0.05). However, the reduction of PI3K and p-Akt was largely suppressed in the mice treated with FELDK after D-gal injection (*P* < 0.05). We did not observe any significant changes in total Akt (Figure 5).

Elevated p-JNK was reported in D-gal-treated mice [6]. Our results suggest that the activation of p-JNK in the D-gal group was comparable to the NC group (*P* < 0.05). FELDK suppressed p-JNK activation in the F1 and F2 groups compared to the D-gal group (*P* < 0.05) (Figure 5).

The results also indicate that D-gal reduced antiapoptotic Bcl-2 and increased proapoptotic Bax compared to the NC group (*P* < 0.01 for both). In contrast, treatment with low and high doses of FELDK reversed the effects on Bcl-2 and Bax protein expression in the cortex and hippocampus of D-gal-aged mice (*P* < 0.05), suggesting that FELDK has antiapoptotic properties (Figure 5).

### 3.7. FELDK Rescues D-gal-Induced Reduction of Synaptic Proteins in the Brain

**Figure 6:**
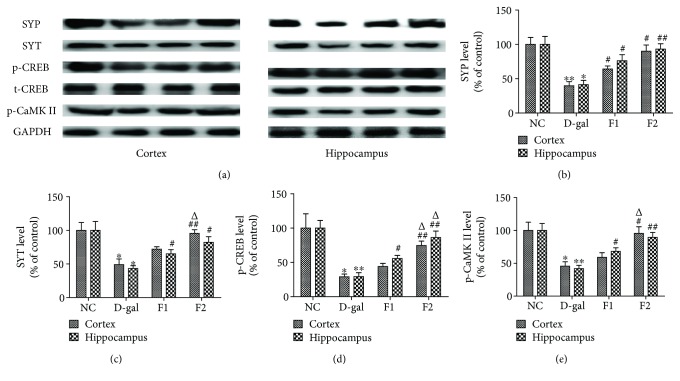
Effects of FELDK on synaptic protein expression, including synaptophysin, synaptotagmin, p-CREB, and p-CaMKII, in the cortex and hippocampus of D-gal-aged mice. Western blot and related quantification of synaptophysin, synaptotagmin, p-CREB, and p-CaMKII protein expression expressed as percent of control. All data are presented as mean values ± SEM for *n* = 3. ^∗^*P* < 0.05, ^∗∗^*P* < 0.01 versus the NC group; ^#^*P* < 0.05, ^##^*P* < 0.01 versus the D-gal group; ^△^*P* < 0.05 versus the F1 group.

Synaptic protein expression was significantly reduced following subcutaneous injection of D-gal [24]. We used western blot analysis to investigate the effect of FELDK on D-gal-induced alterations of synapse-related proteins. The results indicate that synaptic proteins, including synaptophysin, synaptotagmin, p-CREB, and p-CaMKII, were significantly reduced in the D-gal group compared to the NC group (*P* < 0.05). Additionally, FELDK reversed the reduction of synaptic proteins in the F1 and F2 groups compared to the D-gal group (*P* < 0.05) (Figure 6).

## 4. Discussion

Very little is known about the potential antiaging effects of FELDK *in vivo*. Therefore, we investigated the effects and underlying mechanisms of FELDK using the D-gal-aged mouse model. Data obtained from the present study clearly demonstrated the following. First, consecutive subcutaneous injections of D-gal (100 mg/kg/d) daily for 10 weeks resulted in learning and memory deficits, microglia and astrocyte activation, increased AGEs/RAGE and NF-*κ*B nuclear translocation, release of inflammatory mediators, elevated oxidative stress, neuronal cell apoptosis, and dysfunction of synaptic proteins in the brain. Second, oral administration of FELDK (40 or 80 mg/kg/d) significantly attenuated the above-mentioned changes induced by D-gal.

A low dose of D-gal is harmless, as D-gal can be converted into glucose by galactose-1-phosphate uridyltransferase and galactokinase [25]. However, galactose oxidase can increase D-gal and generate ROS [26]. D-gal also reacts readily with amino acids in peptides and proteins to form AGEs, which bind to and upregulate the expression of their receptor, RAGE [9]. ROS and AGEs are both involved in the pathological processes of aging and age-related diseases [27–29]. Studies have demonstrated that chronic subcutaneous administration of D-gal induces ROS and AGEs, which ultimately results in oxidative stress, leading to the activation of glial cells accompanied by neuroinflammation, neuronal cell apoptosis, reduction of synaptic proteins, and memory impairment [6, 14, 15].

ROS and binding of AGEs to RAGE both stimulate NF-*κ*B, which can in turn lead to glial activation and gene transcription of many inflammatory mediators under resting conditions. NF-*κ*B is maintained in the cytoplasm through interaction with the inhibitory subunit I*κ*B. After activation, I*κ*B becomes phosphorylated, followed by polyubiquitination and destruction. As a result, NF-*κ*B is activated and easily translocates from the cytoplasm into the nucleus [30]. Consistent with the previous study, we found that D-gal elevated AGEs, RAGE, Iba1, and GFAP expression, while FELDK reversed AGEs/RAGE signaling and microglia and astrocyte activation. In addition, we demonstrated that D-gal induced nuclear translocation of NF-*κ*B and enhanced the expression of downstream inflammatory markers, including TNF-*α*, IL-1*β*, COX-2, and iNOS. Nevertheless, FELDK attenuated the translocation of NF-*κ*B and release of downstream inflammatory mediators.

Oxidative stress, which is increased in many neurodegenerative diseases, is an important pathological change associated with D-gal-induced aging. Oxidative stress also generates ROS, leading to many other pathological changes that ultimately accelerate the aging process [26]. The degree of oxidative damage can be determined by the production of antioxidant enzymes and markers of oxidative damage products, such as SOD, GSH-Px, CAT, and MDA. SOD combats oxidative stress by converting superoxide anions into H_2_O_2_, a more stable compound [31]. GSH-Px and CAT can further transform H_2_O_2_ molecules into H_2_O [31]. MDA is a pivotal indicator of toxic lipid peroxidation induced by oxidative damage, which indirectly reflects ROS production. It has been reported that FLDK attenuates H_2_O_2_-induced injury in NG108-15 cells by increasing the expression and activity of GSH, CAT, and GSH-Px and reducing MDA levels [17]. In the present study, D-gal-aged mice showed elevated levels of ROS and MDA and decreased activity of SOD, GSH-Px, and CAT compared to the NC mice. However, FELDK markedly reduced ROS and MDA production and upregulated the expression of all measured antioxidative enzymes.

Evidence suggests that apoptosis contributes to the pathological process of D-gal-induced brain injury [32]. PI3K/Akt signaling inhibits cell survival, while JNK activation induces apoptosis through regulating Bcl-2 family gene transcription [33]. In addition, the activation of PI3K/Akt signaling can inhibit the phosphorylation and activation of JNK [34, 35]. Bcl-2 and Bax are members of the Bcl-2 family and function as proapoptotic and antiapoptotic proteins, respectively. Researches have demonstrated that D-gal-induced ROS activates apoptotic signaling via stimulation of JNK in aging animal models [6, 14]. Our findings are consistent with these studies, such that D-gal increased neuronal apoptosis by inhibiting the PI3K/Akt pathway and activating JNK, which ultimately decreased the expression of Bcl-2 and increased the expression of Bax. Previous studies have also shown that FLDK-P70 upregulates Bcl-2 expression in H_2_O_2_-induced NG108-14 cells and protects against hypoxia- and reoxygenation-induced apoptosis *in vitro* [16, 17]. Ethyl acetate extract from the leaves of persimmon significantly attenuated A*β*_1-42_-mediated hippocampus apoptosis in rats by modulating JNK/caspase-3 and the ratio of Bax/Bcl-2 [18]. In the present study, FELDK rescued neuronal apoptosis by upregulating PI3K/Akt and suppressing JNK activation, suggesting that FELDK might possess antiapoptotic activity in the aging mouse brain.

Synaptic proteins correlate closely with cognitive decline and increased oxidative stress [36], providing a good predictor for severity of neurodegeneration [37]. The previous study demonstrated that D-gal reduced the expression of presynaptic and postsynaptic protein markers [9, 24]. Synaptophysin, an important protein that forms small synaptic vesicle membranes, regulates short-term and long-term synaptic plasticity and synapse formation [38, 39]. Synaptotagmin plays a regulatory role in membrane interaction during trafficking of synaptic vesicles at the active zone of the synapse and is involved in exocytosis and endocytosis [40–42]. Autophosphorylation of CaMKII at Thr286 results in its persistent activation and is considered to be pivotal for long-term potentiation and information storage [43]. Phosphorylation of CREB at Ser133 is an important transcriptional process for memory function and synapse formation [8]. Consistent with the previous studies, our data showed that synaptophysin and synaptotagmin, postsynaptic protein p-CaMKII, and p-CREB were all reduced in D-gal-aged mice and oral administration of FELDK markedly enhanced the expression of synaptic proteins, which might be related to improved learning and memory in the aging mice.

**Figure 7:**
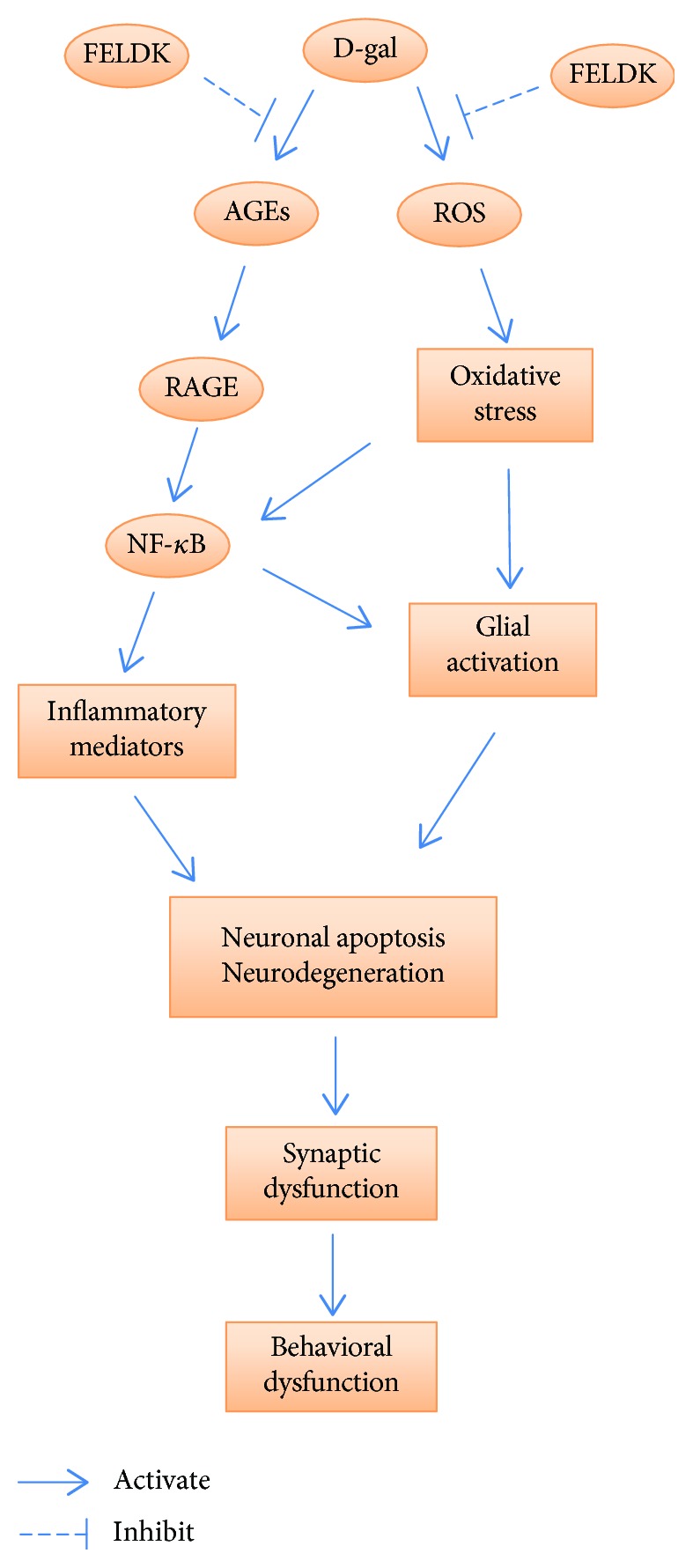
Schematic diagram of the protective effects of FELDK on D-gal-induced brain aging.

In conclusion, FELDK administration alleviated oxidative stress induced ROS production and lipid peroxidation and sustained activity of endogenous antioxidant enzymes. Moreover, FELDK reduced microglia and astrocyte activation, decreased AGEs/RAGE expression, downregulated NF-*κ*B nuclear translocation, inhibited the release of inflammatory mediators, attenuated neuronal apoptosis, and alleviated synaptic dysfunction. As summarized in Figure 7, these effects partially explain the mechanisms by which FELDK attenuates learning and memory impairment in D-galactose-aged senescent mice.

## Data Availability

The data used to support the findings of this study are available from the corresponding author upon request.
